# High-Affinity Glucose Transport in *Aspergillus nidulans* Is Mediated by the Products of Two Related but Differentially Expressed Genes

**DOI:** 10.1371/journal.pone.0094662

**Published:** 2014-04-21

**Authors:** Josep V. Forment, Michel Flipphi, Luisa Ventura, Ramón González, Daniel Ramón, Andrew P. MacCabe

**Affiliations:** 1 Departamento de Biotecnología, Instituto de Agroquímica y Tecnología de Alimentos, Consejo Superior de Investigaciones Científicas (CSIC), Paterna, Valencia, Spain; 2 The Gurdon Institute, University of Cambridge, Cambridge, United Kingdom; 3 Institut de Génétique et Microbiologie, Université Paris-Sud, Orsay, France; 4 Instituto de Ciencias de la Vid y del Vino (CSIC, Universidad de La Rioja, Gobierno de La Rioja), Logroño, Spain; 5 Biopolis SL, Parc Cientific Universitat de València, Paterna, Valencia, Spain; Universidade de Sao Paulo, Brazil

## Abstract

Independent systems of high and low affinity effect glucose uptake in the filamentous fungus *Aspergillus nidulans*. Low-affinity uptake is known to be mediated by the product of the *mstE* gene. In the current work two genes, *mstA* and *mstC*, have been identified that encode high-affinity glucose transporter proteins. These proteins' primary structures share over 90% similarity, indicating that the corresponding genes share a common origin. Whilst the function of the paralogous proteins is little changed, they differ notably in their patterns of expression. The *mstC* gene is expressed during the early phases of germination and is subject to CreA-mediated carbon catabolite repression whereas *mstA* is expressed as a culture tends toward carbon starvation. In addition, various pieces of genetic evidence strongly support allelism of *mstC* and the previously described locus *sorA*. Overall, our data define MstC/SorA as a high-affinity glucose transporter expressed in germinating conidia, and MstA as a high-affinity glucose transporter that operates in vegetative hyphae under conditions of carbon limitation.

## Introduction

Although filamentous fungi pursue largely cryptic lifestyles in the natural environment, these organisms are ubiquitous in terrestrial ecosystems where they play important roles in recycling organic compounds. Along with the capacity to produce an ample spectrum of secondary metabolites [1] and tolerate diverse environmental conditions, their ability as heterotrophs to utilise a wide range of polymeric materials as substrates for growth attests to their metabolic versatility. Since cellular assimilation of the nutrients released from polymers by enzymatic digestion requires their transport across the plasma membrane, the diversity of substrates that filamentous fungi can grow on could reflect the existence of a corresponding variety of uptake mechanisms.

Sugars are the building blocks of plant cell wall polymers and constitute the principal recoverable source of carbon and energy available to fungal saprophytes. The majority of characterised and hypothetical (*i.e.* deduced from genomic sequencing efforts) sugar transporters, be they of prokaryotic or eukaryotic origin, belong to the Sugar Porter (SP) family, one of a large number of protein families defined within the Major Facilitator Superfamily of membrane proteins [2] (http://www.tcdb.org/; TC codes 2.A.1.1 and 2.A.1, respectively). The biochemistry and genetics of sugar uptake in fungi have been most extensively characterised in laboratory strains of *Saccharomyces cerevisiae*. The SP complement of this yeast comprises 34 proteins of which only seven (Hxt1–4, Hxt6, Hxt7 and Gal2) are physiologically relevant transporters of hexoses (Hxt) – their substrates being glucose, fructose, galactose and mannose to varying extents. *HXT* gene expression is subject to control by a regulatory circuit triggered by the sensor proteins Snf3 and Rgt2 (nutrient transceptors) that detect the extracellular concentration of glucose and are themselves closely structurally related to the Hxt proteins [3], [4] (and references therein).

Since the sequencing and annotation of the first *Aspergillus* species (*A. clavatus, A. flavus, A. fumigatus, A. nidulans, A. niger, A. oryzae, A. terreus, Neosartorya fischeri* - http://www.broadinstitute.org/science/data#), genome data for more than 200 filamentous ascomycetes have been acquired (http://www.ncbi.nlm.nih.gov/genome/browse/ and http://genome.jgi.doe.gov/programs/fungi/index.jsf). Bioinformatic identification and assignment of putative sugar permease function (sugar (and other) transporter - PF00083) to genetic loci reveal that the number of such loci per filamentous fungal genome is notably greater than the average of 26 loci observed in an analysis of the SP families of 8 hemiascomycetous yeasts including *S. cerevisiae* [5]. Indeed, more than 100 loci have been assigned to the PF00083 family of *A. nidulans* [6].

Few clues to the identity, function and abundance of sugar transporter genes emerged from classical genetic analyses in *A. nidulans*: four loci (*lacA*, *lacB*, *lacE* and *lacF*) were indicated to be involved in the transport of lactose [7], [8], and mutation at a locus denominated *sorA* was shown to affect the uptake of L-sorbose [9]. Early work measuring the uptake of glucose, galactose and fructose in mycelia [10] provided evidence for the existence of three sugar transport systems, two that were able to transport glucose and galactose but with differing affinities, and a third which was exclusively dedicated to fructose. Similar studies undertaken in *Neurospora crassa* also detected a number of sugar uptake activities including two of high (system II) and low (system I) affinities for glucose as well as a fructose specific system [11]–[14] (and refs therein). Since then [15] two kinetically distinct glucose uptake systems have been identified in wild type germinating *A. nidulans* conidia: a high-affinity system that is expressed in the absence of glucose but repressed in its abundance, and a low-affinity system that is glucose-induced. With regard to the former, high-affinity glucose uptake kinetics were observed to be perturbed in a strain carrying a mutation at the *sorA* locus [15], and this concords with the earlier studies [9] which showed that although mutations at this locus clearly affected the uptake of L-sorbose they also had a minor influence on D-glucose uptake.

Until very recently, the only *A. nidulans* monosaccharide uptake system to have been characterised biochemically, physiologically and genetically was that of the low-affinity glucose transporter encoded by *mstE* - genomic locus AN5860 [16]. Yeast-based functional analysis had been attempted on another putative transporter (HxtA) originally identified in a screen for genes related to sexual development but no sugar transport capability was observed in heterologous expression [17]. In the current report we present an ‘*in situ*’ functional characterisation of two differentially expressed high-affinity glucose transporters (MstA and MstC) after identifying the genes that encode them. One of these corresponds to the glucose repressible glucose uptake system found in germinating *A. nidulans* conidia [15] and is encoded by the gene resident at the classically defined *sorA* locus [9].

## Materials and Methods

### Fungal strains, genetic techniques and culture conditions

The strains used in this work are listed in Table 1. Genetic techniques, culture media and the obtention of conidia for inoculating cultures either for the isolation of RNA or glucose uptake experiments were as described previously [15], [16], [18]. Carbon sources were added to cooled autoclaved media to a final concentration of 1% from filter-sterilised stocks unless stated otherwise. Auxotrophic supplements were added to media where appropriate.

**Table 1:** Fungal strains and genotypes.

Strain	Genotype	Origin
Wild type (V004)	*biA1*	CECT2544
G186	*biA1 sorA2*	[9]; Dr. John Clutterbuck
V045	*pabaA1 sorA3; fwA1*	Prof. H.N. Arst Jr
V048	*pyrG89 pabaA1 yA2*	Dr. Teresa Suárez
V058	*pyrG89 pabaA1 yA2; ΔmstA-pyr4*	This work
LV42	*pyrG89 pabaA1 yA2; ΔmstA-pyr4*	This work
V082	*pabaA1 sorA3; riboB2*	This work
V088	*uaZ11 pabaA1; panB100; riboB2*	This work
V108	*sorA3 yA2; ΔmstA-pyr4; riboB2*	This work
V109	as V088; *ΔmstC-riboB*	This work
V110	as V088; *ΔmstC-riboB*	This work
V111	as V088; ∼*riboB*	This work
V136	as V088; *ΔmstC-riboB*; *ΔmstA-pyr4*	This work
V152	as V136; *mstC_p_-mstA uaZ^+^*	This work

Markers not separated by semi-colons are located on the same linkage group.

The ∼ symbol indicates the presence of the allele in the genome at an unknown location and/or copy number.

Transformations of *A. nidulans* were carried out as detailed previously [19].

Toxic sugar resistance phenotypes were assayed on plates using a medium modified from that originally described by Elorza and Arst [9]. Ethanol (1% v/v) was used as the carbon source rather than glycerol, and 2-deoxyglucose (2-DOG) (50 µg/ml) was the toxic sugar (rather than L-sorbose) since this formulation was observed to give a clearer distinction between *sorA* mutants and wild type: growth of a *sorA^+^* (wild type) strain was completely inhibited whereas that of the *sorA^−^* mutants was unaffected. Where conducted, L-sorbose resistance was tested as detailed by Elorza and Arst [9].

### General molecular techniques

Standard molecular techniques were as described previously [16]. PCR reactions were performed using the Expand High Fidelity PCR System (Roche Applied Science) or DyNAzyme polymerase II (Finnzymes; Espoo, Finland), according to the manufacturers' instructions. Southern blot analysis was carried out using Hybond-N^+^ membranes (Amersham Biosciences); northern blot analysis was performed as detailed [20] using Hybond-N membranes (Amersham Biosciences). Oligonucleotides used in this study are detailed in Table 2.

**Table 2:** Oligonucleotides used in this work.

Oligo name	Nucleotide sequence
M8A	5′-TTCCATTTGGCAGATGTGC-3′
M8S	5′-CGTGGTTGAACTGCTTCC-3′
spMstAIII3′	5′-GTGTGATAATTCCAAAG-3′
mstA8	5′-GTGTCGGTTTTGTCTCTGCG-3′
M8A	5′-CGAATTCCATTTGGCAGATGTGC-3′
spMstAI	5′-GCGTAAATGTACATTTTTGG-3′
mstA(LI)3′-2	5′-CAATCCAACGACATGAAGC-3′
LK1A1	5′-CCCAAGCTTGAATTCGCCATCCGTAAC-3′
LK1B2	5′-GGAATTCGCTGTCTTGTTCAATC-3′
AI4	5′-GGAAGTGCTTAAGCAGTGGATTCTCTCTTTCCTGCGGTTTCACTTCGGTATCGCATTTG-3′
AI8	5′-GCAAGGACCACATTATCTTGCCCACCCCTCCGTATTGGCGTGAACAAAATTGCCTCTAGTCCTTGG-3′
R3	5′-CAAATGCGATATCCGAAGTGAAACCGCAGGAAAGAGAGAATCCACTGCTTAAGCACTTCC-3′
R5	5′-GGACTAGAGGCAATTTTGTTCACGCCAATACGGAGGGGTGGGCAAGATAATGTGGTCCTTGC-3′
mstA(I)p5′	5′-CCCAAGCTTCGGACGTAAACAGAATGGCGTGTTTGACAGG-3′
mstA(III)-mstA(I)p3′	5′-CACGCGCTGACCAGCAATCACCGCGTCTGCCATGGGCGAGGGGGCGTGAAGGTGACGCGGTAGG-3′
mstA(I)p-mstA(III)5′	5′-CCTACCGCGTCACCTTCACGCCCCTCGCCATGGCAGACGCGGTGATTGCTGGTCAGCGCGTG-3′
mstA(III)t3′	5′-CTACCAAGCTTAGAGAAGCTAGCTCCCGGCAACAATGACC-3′
H3N-mstA(I)p5′	5′-CCCCCCAAGCTTGTTTGACAGGTAAGAG-3′
H3N-mstA(III)t3′	5′-CCCCCCAAGCTTCACACTCTATGCATGC-3′
mstA(I)t3′	5′-CCCAAGCTTCAATCACAAAGGACAACACAGTGTGTACG-3′
mstCwt	5′-CTGGGGTCCCGGTGCCT-3′
A1Eco	5′-CCCAAGCTTGAATTCGCCAATGTTTGG-3′
A2	5′-CGGGATCCTACATGGAGGAGAACG-3′
B1	5′-CGGGATCCGAATACAGCAAGGATG-3′
B2	5′-GGAATTCTGCGTATCTGAGTGC-3′
mstAc5′	5′-TCGAATTCTGATCTCGCGCTCACTGAG-3′
mstAc3′	5′-CCGCTCGAGGACATCCTTGCTGTATTCC-3′
chekF	5′-GATGTTTGCGGCACGTTATTGGCAGG-3′
chekR2	5′-TGATAGCACCACCTAGTAGTCATGGCAG-3′
LKIA3	5′-GTGCAAGACCAAGCGAG-3′
mstA11	5′-TGATAAGGGATTTATTCGAC-3′

### Probes

The probe specific for the *mstA* coding sequence identified from EST overlaps was generated by PCR off an *A. nidulans* wild type genomic DNA (gDNA) template using oligonucleotide primers M8A and M8S. This was used to screen a λZAP-based *A. nidulans Bam*HI genomic library (partial) kindly provided by Prof. Claudio Scazzocchio.

Probes specific for the *mstA* and *mstC* transcripts were prepared by PCR and correspond to the 3′ untranslated regions immediately following the stop codons of each coding sequence - the level of nucleotide sequence identity shared between these regions is similar to that observed for unrelated sequences. The *mstA* probe was primed off oligonucleotides B1 and spMstAIII3′ and yielded a ∼200 bp fragment; the *mstC* probe was primed off oligonucleotides spMstAI and mstA(LI)3′-2 yielding a ∼150 bp fragment.

A 4.7 kb DNA fragment corresponding to the genomic locus of the *mstC* gene which was used to cotransform *A. nidulans* strain V082 was amplified off an *A. nidulans* wild type gDNA template using oligonucleotides mstA(I)p5′ and mstA(I)t3′. The resulting product was sequenced and no mutations were detected. Cotransformation of V082 was done using 3 µg of the *mstC* fragment together with 2 µg of plasmid pPL5 [21] that carries the *A. nidulans riboB* gene and complements the *riboB2* mutant allele. The presence of the wild type (transforming) *mstC* gene was detected by conducting PCR on gDNA isolated from riboflavin prototrophic transformants using the oligonucleotide primers mstCwt and mstA(LI)3′-2. mstCwt was specifically designed to amplify only the wild type *mstC* gene and not the point-mutated allele present in the *sorA3* genetic background. When used in conjunction with mstA(LI)3′-2 and a PCR annealing temperature of 67°C a 550 bp fragment is produced in the presence of the wild type *mstC* gene. No product is yielded by the *mstC* mutant allele under these conditions.

### Gene deletion

The *mstA* gene: plasmid pPTmstApyr4 was engineered to contain a deletion cassette comprising the *N. crassa pyr4* gene [22] (a 3.25 kb *Bgl*II fragment) flanked by ∼1 kb of PCR-generated sequences corresponding to the upstream (oligonucleotides A1Eco and A2) and downstream (oligonucleotides B1 and B2) regions of the *A. nidulans mstA* gene. The cassette (∼5.25 kb) was excised by *Eco*RI digestion and used to transform *A. nidulans* strain V048. gDNA was isolated from clonally purified uridine prototrophic transformants and used as template in PCRs with the oligonucleotides mstAc5′ and mstAc3′ that amplify a 1.9 kb *mstA*-specific fragment. The absence of this product was taken to be indicative of the deletion of *mstA*. Confirmation of *mstA* deletion was done by probing Southern blots of *Bam*HI- or *Hin*dIII-digested gDNAs with a PCR-generated probe (oligonucleotide primers mstA8 and M8A) corresponding to the central structural sequence of *mstA*. The 2.4 kb *Bam*HI fragment present in the non-transformed strain (control) was absent in the deletion mutants.

The *mstC* gene: double-joint PCR [23] was employed to construct a linear DNA fragment comprising the *A. nidulans riboB* gene [21] (oligonucleotides R5 and R3) flanked by the *mstC* upstream (oligonucleotides LK1A1 and A18) and downstream (oligonucleotides A14 and LK1B2) genomic sequences (∼3.5 kb of each). This linear construct was used to transform *A. nidulans* strain V088 and selection was done on appropriately supplemented medium (lacking riboflavin) containing D-sorbitol as both carbon source and osmotic stabilizer. Transformants (∼300) were subsequently clonally purified on plates containing glycerol as the carbon source. All were assessed for their resistance to 2-DOG and L-sorbose by plate testing resulting in the identification of nine 2-DOG-resistant transformants. gDNA samples isolated from each of these resistant clones as well as a randomly chosen transformant that remained sensitive to 2-DOG were used as templates in PCRs with primers (chekF and chekR2) designed to yield a product (∼840 bp) only in the case of replacement of the *mstC* gene by *riboB*. All gDNAs derived from 2-DOG-resistant transformants yielded the 840 bp PCR fragment whereas that from the 2-DOG-sensitive transformant failed to do so. Southern analyses carried out on several *Bam*HI-digested gDNAs using probes specific for the *riboB* and *mstC* genes also confirmed the elimination of *mstC* and its substitution by *riboB* in 2-DOG-resistant transformants.

### Construction of a strain expressing *mstA* during germination

An *A. nidulans* strain (V136) carrying the *uaZ11* [24] mutation and deleted for both the *mstA* and *mstC* genes was obtained from a sexual cross between strains V108 and V109. Strain V136 was subsequently transformed with a *uaZ11*-complementing plasmid (‘p*uaZ*-petit’ - carries the 2 kb *Cla*I/*Xho*I fragment essential for site-directed integration in the *uaZ11* mutant allele; kindly provided by Dr. Béatrice Felenbok) into which had been inserted a translational fusion (made by overlap extension PCR) between PCR-generated fragments corresponding to the *mstA* structural gene (2.4 kb) and the 1.9 kb of sequence immediately upstream of the *mstC* translational initiation codon. The *mstA* coding sequence and the *mstC* promoter fragments used for this were generated off the oligonucleotide primer pairs mstA(I)p-mstA(III)5′ and mstA(III)t3′, and mstA(I)p5′ and mstA(III)-mstA(I)p-3′, respectively. The fusion product was ultimately recovered by PCR using the nested oligos H3N-mstA(III)p5′ and H3N-mstA(III)t3′, and inserted into p*uaZ*-petit using *Hin*dIII. DNA sequencing confirmed the absence of mutations in the fragments used to make the construct and in the final construct itself. The strong bias for the selection of single copy integration of the transforming DNA at the *A. nidulans uaZ* locus conferred by the restored ability to grow on uric acid (100 µg/ml) as sole nitrogen source resulted in the identification of a transformant (V152) in which the presence of the *mstC_p_-mstA* fusion was confirmed by the specific amplification of a 645 bp PCR fragment off a genomic DNA template using the primers LKIA3 (located on the sense strand of *mstC_p_*) and mstA11 (located on the antisense strand of *mstA*). The genomically integrated fusion construct was also sequenced in its entirety revealing the absence of mutations.

### Glucose uptake experiments

Glucose uptake experiments were performed as described previously [16]. All sugars used were in the ‘D’ configuration unless stated otherwise. Glucose uptake rates in substrate competition experiments were calculated from measurements of the amount of glucose taken up in 5, 30, 60 and 90 seconds (done in quadruplicate); competing compounds were present at 200 fold molar excess (*i.e.* 3.2 mM and 18 mM for V004 and V152, respectively) and the ^14^C-glucose concentration for each was fixed at 16 µM and 90 µM respectively.

To assess energy requirements, glucose uptake by glycerol-germinated conidia was measured at 30, 60 and 90 seconds (each time point was done in triplicate) in the presence and absence of 30 µM carbonyl cyanide m-chlorophenylhydrazone (CCCP) at glucose concentrations of 16 µM for V004 and 90 µM for V152. Uptake rates were derived from the slopes of the plots obtained.

All data were processed using SigmaPlot 12.

### Informatic analysis

Transporter protein primary structures were aligned with MUSCLE [25], [26] and the best amino acid substitution model (LG+I+G) was determined using Prottest3 [27], [28]. The unrooted maximum likelihood phylogeny tree was generated using PhyML [29] and drawn using MEGA5 [30]. The data set used is available on request.

## Results

### Identification of the *A. nidulans mstA* and *mstC* genes encoding putative sugar transporters

**Figure 1:**
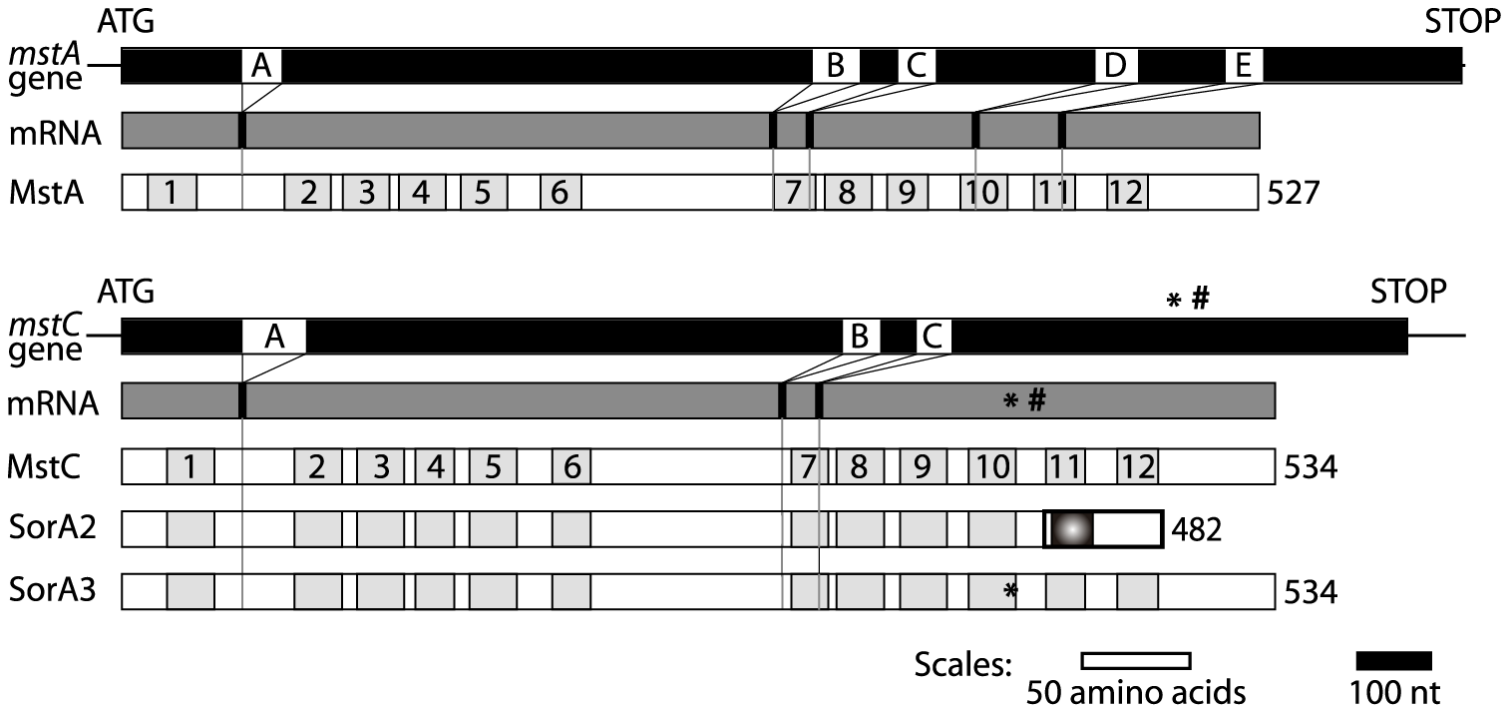
Structural organisation of the *mstA* and *mstC* genes and their translation products. The upper bars are schematic representations of gene structure with introns (A to E) in white and exons in black. cDNAs generated by RT/PCR from transcripts (dark grey bars) of each gene were sequenced and compared with the genomic sequences, thus confirming the intron/exon structures deduced for each gene. Proteins are shown as white bars within which the numbered grey-shaded boxes correspond to the TM domains predicted by TMHMM [31]. The locations of the mutations and the corresponding changes in the 1° structure of MstC found in the *sorA2* (#) and *sorA3* (*) mutants are also shown (see text for details). The *sorA2* mutation causes a change in reading frame resulting in a shorter and novel COOH-terminal sequence (shown in bold) within which resides a putative TM domain (marked with a circle). The annotation of the *A. nidulans* genome assigned the locus identities AN8737 and AN6669 to *mstA* and *mstC*, respectively.

A set of overlapping EST sequences corresponding to a candidate sugar transporter gene (designated *mstA*) were originally identified in the Oklahoma University *A. nidulans* EST database (http://www.genome.ou.edu/fungal.html). A PCR-generated probe based on this data was used to clone the corresponding genomic sequence from an *A. nidulans* λZAP *Bam*HI genome library and a hypothetical gene comprising six exons encoding a putative twelve transmembranal (TM) domain protein of 527 amino acids (MstA) was deduced (Figure 1; GenBank AJ251561).

**Figure 2:**
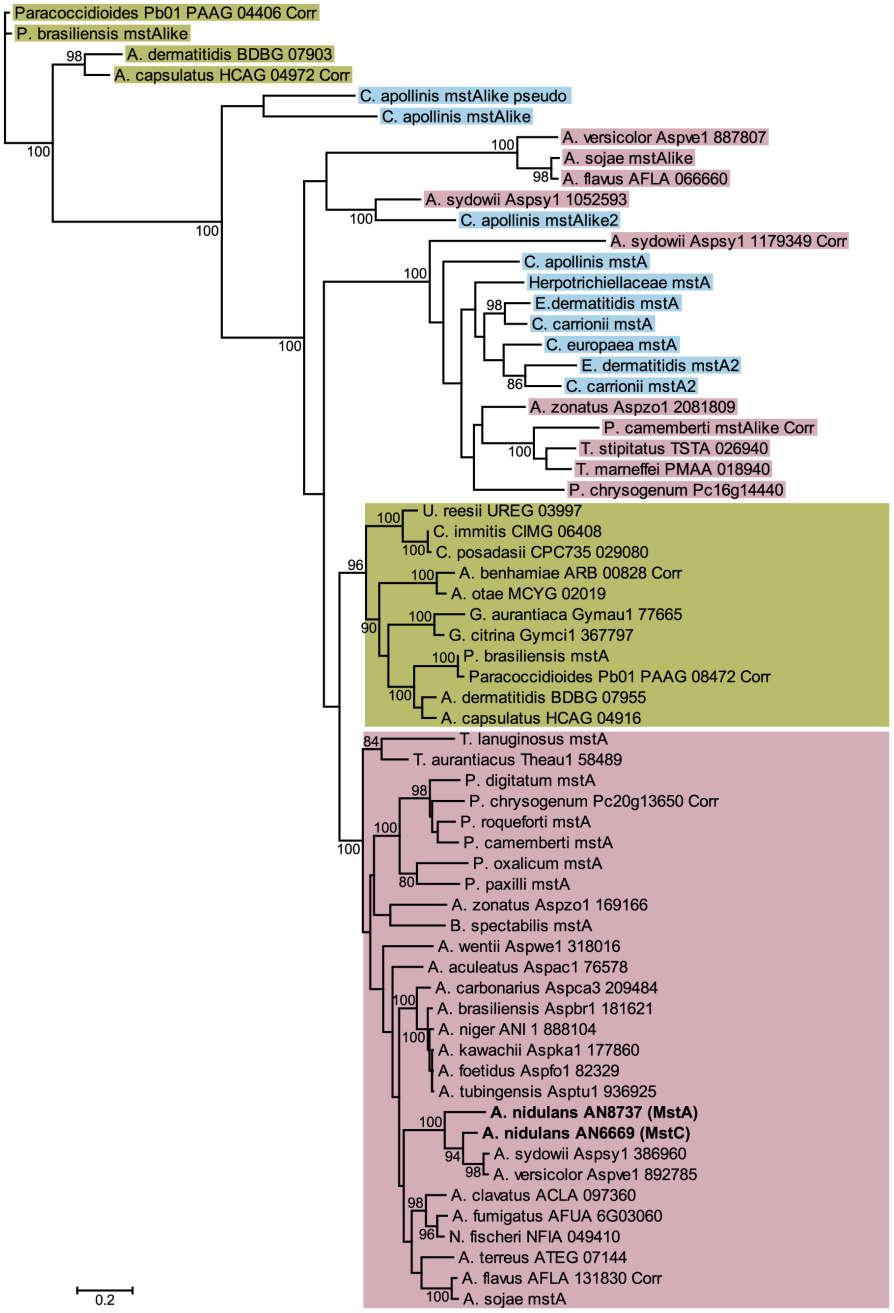
Unrooted phylogenetic tree of primary structures of Eurotiomycete proteins related to MstA (AN8737) and MstC (AN6669). The evolutionary history was inferred using the Maximum Likelihood method, and the percentages of replicate trees in which the associated sequences clustered together in the bootstrap test (50 replicates) are shown next to the branches (values below 80% are not included). Branch lengths correspond to the mean number of substitutions per site. Where known, genome locus identities are given; ‘Corr’ indicates that the gene model was corrected; unannotated sequences are given as ‘mstA-like’. The homologues in *A. flavus* and *A. oryzae* were found to be encoded by identical genomic DNA sequences (only *A. flavus* is shown), as expected for organisms that are believed to be variants of the same species [33]. *A. nidulans* is the only organism represented that possesses two very closely related proteins. MstA (AN8737) and MstC (AN6669) are shown in bold.

Once available, the high coverage (×13) *A. nidulans* genome sequence [32] was screened (TBLASTN) using the primary structure of MstA revealing the existence of a closely related putative translation product of 534 amino acids (84% identity, 91% similarity). The potential coding sequence deduced (designated *mstC*; GenBank AJ879992) comprises four exons (Figure 1) and is identical to that determined by the *A. nidulans* genome sequencing project [32]. Low stringency Southern analysis (not shown) confirmed cross-hybridisation between the *mstC* gene and a probe corresponding to the whole *mstA* structural gene, and *vice versa*. BLAST searches of the PRINTS and PFAM databases using the primary structures of MstA and MstC support their designation as members of the sugar transporter family (PR00171; PFAM: PF00083), and TBLASTN searches of the sequence databases (NCBI, The Broad Institute and the Joint Genome Institute) identified a number of similar Eurotiomycete proteins. A phylogeny tree of these is presented in Figure 2. Two major clades emerge which correspond to the homologues present in the orders Eurotiales (pink) and Onygenales (yellow). Whilst a few of these species and some members of the order Chaetothyriales (blue) have one or more distant paralogues, *A. nidulans* is unique in possessing two paralogues that are so closely related.

The assembly of the *A. nidulans* genome made it possible to correlate the resulting physical maps of the chromosomes with the linkage maps established from decades of classical genetic analysis [34]. The *mstA* gene was thus assigned to chromosome III but it was of considerably greater interest to find that the *mstC* gene resides in a region of chromosome I (contig 1.110) to which mutant *sorA* alleles have been genetically mapped, since the corresponding mutants had previously been found to manifest an altered sugar transport phenotype [9]. With regard to the latter, more recent studies have confirmed a role for the *sorA* locus in high-affinity glucose uptake in germinating conidia [15]. In view of this, the *mstC* gene was sequenced in two different *sorA* mutants and also in several *sorA* ^+^ strains available in our laboratory. Whilst the latter yielded *mstC* sequences identical to that found by the genome sequencing project, the *sorA* mutants yielded discrepancies. In one strain (V045) carrying the mutant allele *sorA3* (GenBank AJ879993) a missense mutation was found that would result in a W412R replacement in TM domain 10 (Figure 1); in the *sorA2* mutant (strain G186) [9] an 11 base pair sequence duplication (GenBank EU366284) located between TM domains 10 and 11 would result in a shortened translation product (482 amino acids compared to 534) in which the C-terminal 58 amino acids are derived from a different reading frame and hence are unrelated to the wild type sequence of MstC (Figure 1). The coincidence of the genetic locations of *sorA* and *mstC* in conjunction with the observation that two independent *sorA* mutant strains also carry non-identical mutations in the *mstC* gene is indicative of allelism between *sorA* and *mstC*.

### *mstC* encodes a high-affinity glucose transporter expressed in germinating conidia

#### i) Marker rescue of *sorA3* by *mstC*

**Figure 3:**
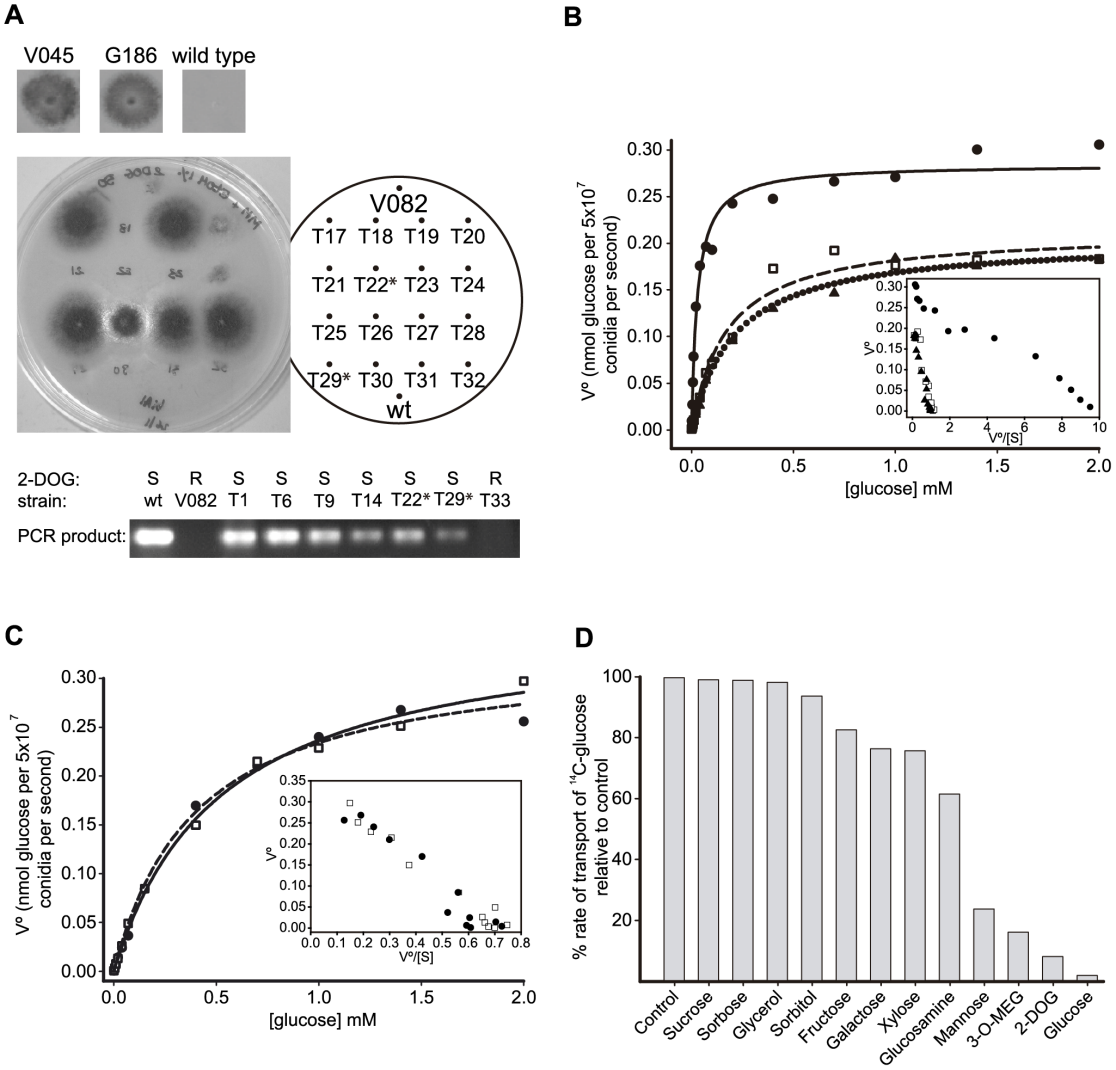
Characterisation of MstC. **A** Marker rescue of a *sorA* mutant by *mstC*. Top panel: *sorA* mutant strains (V045 and G186) are able to grow in the presence of 50 µg/ml 2-DOG and 1% EtOH compared to wild type. Middle panel: A typical minimal medium plate lacking riboflavin but supplemented with 2-DOG used for identifying 2-DOG sensitive transformants (those that do not grow); V082 fails to grow as it is auxotrophic for riboflavin. Lower panel: typical PCR products specific to the wild type *mstC* allele amplified off gDNA templates. R = resistant to 2-DOG, S = sensitive to 2-DOG. T = transformant. T22 and T29 are asterisked to help their identification on the plate in the middle panel. T33 is a control transformed with pPL5 alone. **B** Typical Michaelis-Menten plots of glucose uptake rate *versus* glucose concentration for conidia of the two *ΔmstC* strains V109 (□) and V110 (▴), and the *mstC^+^* strain V111 (•) germinating for 4 h in appropriately supplemented minimal medium containing 1% glycerol as carbon source; non-linear regressions are shown as dashed, dotted and solid lines, respectively. Insert: Eadie-Hofstee plots. The plots for V109 and V110 are monophasic. Their displacement towards the y axis relative to the plot of V111 is indicative of the loss of high-affinity uptake. **C** Typical Michaelis-Menten plots of glucose uptake rate *versus* glucose concentration for conidia of strains V140 (*sorA2*) (□) and V045 (*sorA3*); non-linear regressions are shown as solid and dashed lines, respectively. (•) germinating for 4 h in appropriately supplemented minimal medium containing 1% glycerol as carbon source. The insert shows Eadie-Hofstee plots of the uptake data for both strains. **D** Relative ^14^C-glucose uptake rates in the presence of a 200-fold molar excess of competing compounds are expressed as a percentage of the non-competed (control) rate.

Since mutant *sorA* alleles confer resistance to toxic sugars such as L-sorbose and 2-deoxyglucose (2-DOG) [9], successful phenotypic rescue of a *sorA* mutation should result in restored sensitivity to these compounds. To test the possibility that *mstC* could rescue mutation at the *sorA* locus, the 2-DOG resistant *sorA3* strain V082 (also auxotrophic for riboflavin) was cotransformed with plasmid pPL5 [21] (provides the *riboB* selectable marker) and a 4.7 kb PCR-generated linear DNA fragment comprising the entire *mstC* structural gene including the upstream (1.9 kb) and downstream (0.95 kb) flanking sequences. Almost half (seventeen) of the riboflavin prototrophic transformants obtained (forty) were found to have gained sensitivity to the presence of 2-DOG (Figure 3A - top and middle panels), whereas a control transformation using pPL5 alone yielded none. PCR analyses were subsequently carried out on a number of randomly chosen cotransformants using an oligonucleotide primer pair designed to amplify only the wild type *mstC* gene but not the mutated allele found in the *sorA3* mutant (see Materials and Methods). The predicted amplification product was present uniquely in those transformants that exhibited restored sensitivity to 2-DOG (Figure 3A - lower panel). Neither the untransformed strain (V082) nor the randomly chosen transformants that maintained resistance to 2-DOG yielded it. Thus the *mstC* gene is able to effect marker rescue, restoring a *sorA* mutant strain to 2-DOG sensitivity.

#### ii) *mstC* gene deletion and phenotype

The evidence for allelism of *sorA* and *mstC* raises the possibility of the involvement of the latter in high-affinity glucose uptake. A gene replacement strategy was therefore employed to delete *mstC*, substituting it with the *riboB* gene [21] in a riboflavin auxotrophic strain (V088) - details are given in Materials and Methods. Two phenotypic characteristics were examined in the *ΔmstC* transformants generated, namely their resistance/sensitivity to toxic sugars and the kinetics of glucose uptake by their germinating conidia.

- *Toxic sugar resistance:* Since the enhancement/absence of high-affinity glucose uptake has been seen to correlate with greater sensitivity/resistance (respectively) to toxic sugars [9], [15], all V088-derived transformants (riboflavin prototrophs) were plate assayed for their sensitivity/resistance to 2-DOG (50 µg/ml). Of more than 300 prototrophic transformants obtained, nine exhibited enhanced resistance to this compound and were phenotypically indistinguishable from the *sorA3* control. The members of this subset were also found to be resistant to L-sorbose, and PCR analysis demonstrated the absence of the *mstC* gene in all of them. Southern blot analysis carried out on four randomly chosen 2-DOG resistant transformants confirmed that the deletion of the *mstC* gene was the result of a single reciprocal integration of the *riboB* gene at the *mstC* locus (data not shown). Riboflavin prototrophic transformants in which the *mstC* gene was unaltered (*i.e.* the transforming DNA had integrated elsewhere in the genome) failed to show enhanced resistance to the toxic sugars. As regards their growth and morphology either on plates or in liquid culture in which glucose was present as sole carbon source, the *mstC*-deleted transformants did not present a visible phenotype distinguishable from that of non-deleted strains.
- *Glucose uptake:* Using culture conditions that have been shown to result in high-affinity glucose transport in germinating (4 h) wild type conidia (*i.e.* glycerol as carbon source) [15], ^14^C-glucose uptake was measured in two *ΔmstC* transformants (V109 and V110) as well as in a transformant (V111) complemented for the *riboB2* allele but in which the *mstC* gene had not been deleted (see above) and hence acts as a suitable control. Visual monitoring by light microscopy failed to detect any differences in the germination process between the gene-deleted and the non-deleted strains. Whereas Eadie-Hofstee representation and non-linear regression analysis of the Michaelis-Menten plot of the control strain (V111) revealed high-affinity glucose uptake kinetics (Km ∼30 µM), both *ΔmstC* transformants showed considerably reduced uptake affinity, yielding Km values (∼300 µM) similar to that previously reported for the *sorA3* mutant [15] (Figure 3B). The loss of high-affinity glucose transport in the *ΔmstC* strains is thus indicative of a role for *mstC* in this uptake system.

### Glucose uptake in a *sorA2* mutant

That the kinetics of glucose uptake by *ΔmstC* (*i.e.* total loss of MstC function) strains are essentially the same as those of the *sorA3* mutant strongly indicates that the *mstC* point mutation present in the latter results in total loss of MstC function. It is therefore reasonable to expect that the frameshift mutation found in the *sorA2* mutant (see above) that results in a much greater modification of MstC primary structure (20% of the protein is altered at the C-terminus) compared to *sorA3* would also result in total loss of function.^14^C-glucose uptake was measured and compared between *sorA2* (V140) and *sorA3* (V045) conidia germinating in parallel cultures under identical conditions that induce the high-affinity glucose uptake system (1% glycerol). The Michaelis-Menten kinetics of glucose uptake for both strains were practically indistinguishable (Figure 3C), and the Eadie-Hofstee representations of each are consistent with the same monophasic behaviour. These data imply similar consequences of the two *sorA* mutations on the high-affinity glucose transport function and provide further evidence for allelism of *sorA* and *mstC*.

### MstC substrate assay

In order to assess the potential substrate range of the high-affinity glucose uptake system, substrate competition experiments were conducted using glycerol-germinated wild type conidia. ^14^C-glucose uptake rates were measured in the presence of a 200-fold molar excess of individual compounds under test and compared to that of a control assay performed in the absence of a competing substrate. The data obtained are summarised in Figure 3D. Glucose (non-radiolabelled) was found to be the most effective competitor, along with the glucose analogues 2-DOG and 3-O-methyl glucose. Mannose also demonstrated competition resulting in a reduction of uptake by 80%. Far less competition was exercised by galactose, xylose and fructose suggesting that these sugars could be only very minor substrates. A number of other compounds were tested none of which demonstrated effective competition. These data thus show that of the range of compounds tested, glucose is the principal substrate of the uptake system that is expressed in glycerol-germinating wild type conidia.

### *mstA* encodes a high-affinity glucose transporter

**Figure 4:**
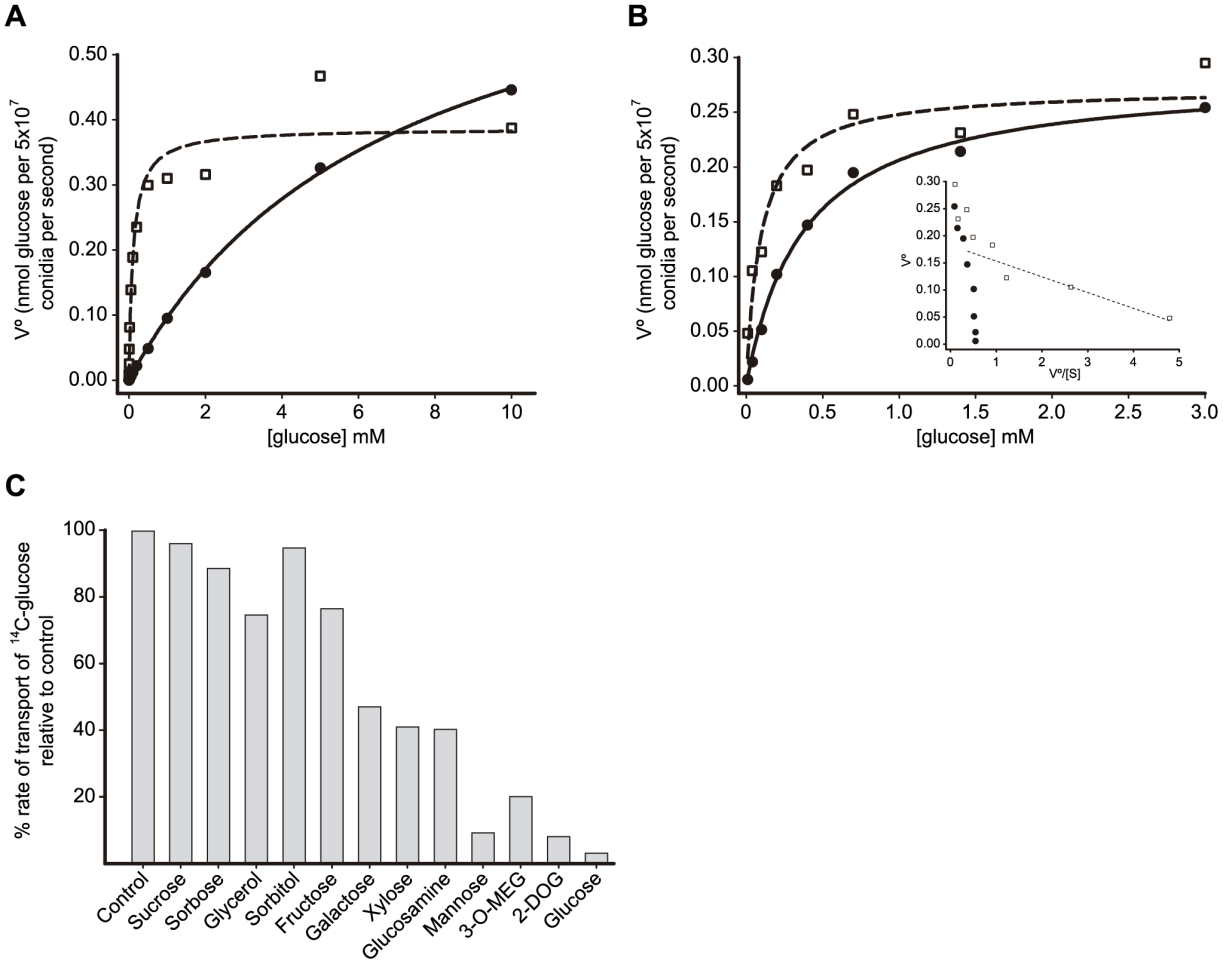
Characterisation of MstA. **A** Typical Michaelis-Menten plots of glucose uptake rate *versus* glucose concentration for *ΔmstA* conidia germinating for 4 h in appropriately supplemented minimal medium containing either 1% glycerol (□, dashed line) or 1% glucose (•, solid line) as carbon source. **B** Typical Michaelis-Menten plots of glucose uptake rate *versus* glucose concentration for conidia of strains V136 (•) and V152 (□) germinating in appropriately supplemented medium containing 1% glycerol as carbon source. The respective solid and dashed lines correspond to the non-linear regressions from which the Km values were obtained. The insert shows Eadie-Hofstee plots of the uptake data for both strains. The dashed line corresponds to the higher affinity uptake component present in strain V152, the slope of which yields an approximation to the Km of this component (∼30 µM). **C** Relative ^14^C-glucose uptake rates in the presence of a 200-fold molar excess of competing compounds is expressed as a percentage of the non-competed (control) rate.

Given the evidence accumulated supporting a direct relationship between the *sorA* locus, the *mstC* gene and high-affinity glucose uptake, attention was turned to identifying a function for the closely related gene *mstA*. A gene deletion protocol was used to substitute *mstA* with the *N. crassa pyr4* gene in the uridine auxotrophic strain V048, and the kinetics of glucose uptake were subsequently determined for the conidia of two independently obtained *mstA*-deleted transformants (V058 and LV42; *mstA* deletion was confirmed by PCR and by Southern blotting) germinating in appropriately supplemented minimal medium containing either glucose or glycerol as sole carbon sources. Glucose uptake kinetics by the *ΔmstA* strains were found to be the same as those observed for wild type conidia: low-affinity kinetics upon germination on glucose, and high-affinity uptake kinetics when germinating on glycerol (Figure 4A). This is in notable contrast to the glucose uptake phenotype of the *mstC* deletion strains (see above) and indicates that the *mstA* gene does not play a significant role in glucose uptake during conidial germination.

The absence of function of MstA in germinating conidia and the considerable similarity shared between the primary structures of MstC and MstA (91% - see above) could be indicative of a glucose transport function for MstA that is unrelated to the germination process. To examine this possibility an *A. nidulans* transformant (strain V152) was generated in which a single copy of the *mstA* structural gene under the control of the *mstC* gene promoter (*mstC_p_*) was placed at the *uaZ* locus in a genetic background deleted for both the *mstC* and *mstA* loci (strain V136) - details are given in Experimental Procedures.

Prior to undertaking glucose uptake measurements, an indication of the functional competence of the *mstA* gene product under the control of *mstC_p_* was revealed by plate-testing the 2-DOG sensitivity of V152 (*ΔmstA ΔmstC mstC_p_-mstA*) compared to that of controls comprising a wild type strain (*mstA^+^ mstC^+^*), the double mutant V136 (*ΔmstA ΔmstC*) and the *sorA3* mutant V045 (*mstC^−^*). Unlike V045 and V136, strain V152 displayed wild type sensitivity to 2-DOG indicating that acquisition of the *mstA* expression construct resulted in restored sensitivity to 2-DOG, presumably by virtue of restoration of a glucose uptake function (data not shown).

To assess a possible glucose transport function for MstA, ^14^C-glucose uptake was measured across a range of glucose concentrations (Figure 4B) in conidia of strains V136 (*ΔmstA ΔmstC*) and V152 (*ΔmstA ΔmstC mstC_p_-mstA*) germinating for 4 h in glycerol medium - conditions that induce high-affinity uptake. Using non-linear regression, the Km of glucose uptake by the doubly-deleted mutant (V136) was found to be ∼350 µM, a value corresponding to that previously observed for the *ΔmstC* strains (V109 and V110) and the *sorA* mutants (V045 and V140). V152 in contrast yielded a Km of ∼90 µM. The uptake data for both strains were also plotted using the Eadie-Hofstee representation. Whilst the distribution of data points observed for V136 (•) is indicative of single component behaviour (*i.e.* compatible with a straight line), the non-linear nature of the plot for V152 (□) is consistent with the presence of an additional component of higher glucose affinity (indicated by the dashed line in the insert). Whilst bearing in mind the known limitations of the approximation [35], [36], the gradient modulus of the line defined by the four data points of lowest V° values yields an orientative Km of around 30 µM for this element, a value very similar to that calculated for MstC-mediated glucose transport (see above). These data thus support a glucose uptake function for MstA and indicate that MstC and MstA share comparable glucose affinities.

In the same way as undertaken for MstC (see above), the substrate range of MstA was also assessed using glycerol-germinated conidia of strain V152. The data obtained are shown in Figure 4C. Whilst some influence of the minor low-affinity component of uptake by V152 cannot be excluded, this analysis indicates that mannose is the most effective substrate of those studied in competing against glucose uptake, and that xylose and galactose may also be minor physiological sugar substrates for this transporter.

### Energetics of high-affinity glucose uptake

**Figure 5:**
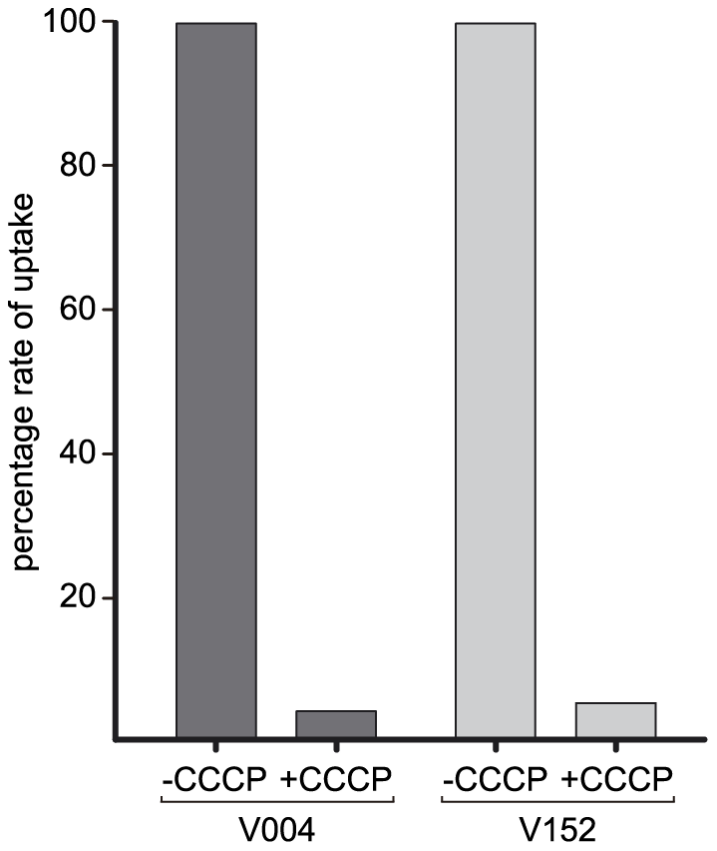
Energetics of glucose uptake. Relative glucose uptake rates for glycerol-germinated conidia expressing MstC (dark grey - V004) and MstA (light grey - V152) transporters assayed in the absence (−) and presence (+) of CCCP.

It has been shown previously [15] that glucose uptake by both high- and low-affinity systems in *A. nidulans* are energy-requiring processes. Carbonyl cyanide m-chlorophenylhydrazone (CCCP) is a powerful uncoupling agent that leads to rapid loss of membrane potential [37]. The effect of CCCP on glucose uptake was therefore assessed in conidia expressing MstC (glycerol-germinated V004) and those expressing MstA (glycerol-germinated V152). Uptake rates for both were dramatically reduced in the presence of CCCP (Figure 5) thus strongly suggesting MstC and MstA to be secondary active transporters.

### *mstA* and *mstC* expression

Increased resistance to toxic sugars and the loss of high-affinity glucose transport have both been seen to be phenotypic consequences of *mstC* deletion. Whilst no such phenotypes were forthcoming upon deletion of *mstA*, expression of the *mstA* structural gene off the *mstC* gene promoter in a *ΔmstA ΔmstC* genetic background did result in the reciprocal phenotypes (*i.e.* sensitivity to toxic sugars and the presence of high affinity glucose uptake - see previous section). Given that earlier observations have demonstrated that the nature of the glucose uptake system expressed in germinating conidia is regulated in response to the carbon source available [15], [16], the apparent lack of a *ΔmstA* phenotype could be due to differences in the expression profiles of the *mstC* and *mstA* genes.

**Figure 6:**
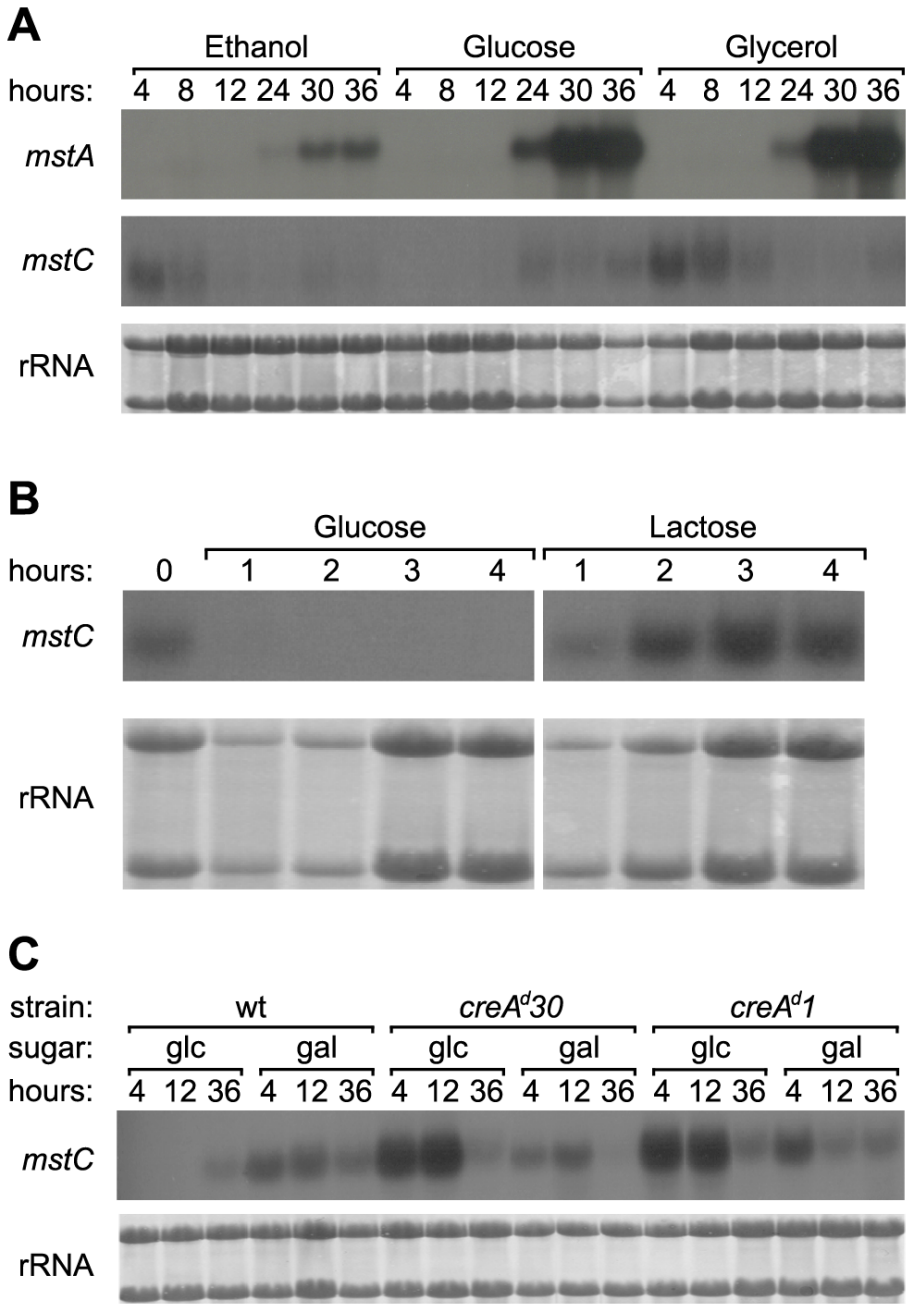
Northern analyses of gene expression. **A** Total RNA was isolated from shake flask biomass grown in the presence of the following carbon sources (initial concentration): ethanol (0.5% v/v) a poor, derepressing source; glucose (0.5% w/v) a good, repressing source; and glycerol (0.5% w/v) a good, derepressing source. All sources were exhausted by 20 h. Biomass was harvested at the times indicated after inoculation of conidia. **B** *mstC* mRNA accumulation during conidial germination in media containing glucose or lactose (each present at an initial concentration of 0.5% w/v). Total RNA was isolated from biomass harvested from shake-flask cultures at the times indicated after inoculation of conidia. **C** *mstC* mRNA accumulation in two CCR mutants (*creA^d^*) compared to wild type (wt). The two CCR mutants and the wild type strain were grown in the presence of glucose (glc) or galactose (gal), each initially present at 0.5% (w/v). Total RNA was isolated from biomass harvested from shake-flask cultures at the times indicated after inoculation of conidia. In all cases cultures were grown from conidia for the times indicated, and rRNA was visualised by methylene blue staining [39].

In order to investigate the possible influence of carbon source, expression of *mstA* and *mstC* was assessed by northern blot analysis. Total RNA was isolated from wild type *A. nidulans* mycelia obtained after various periods of growth from conidia inoculated into liquid growth medium containing carbon sources of diverse metabolic properties (ethanol, glucose or glycerol). The latter refers to whether on the one hand the carbon source is considered to be a relatively good or a relatively poor substrate for the growth of the micro-organism, and on the other whether it is classified as being repressing or derepressing as determined by its impact on the utilisation of L-proline and acetamide as nitrogen sources in *areA^r^* mutants [38]. Given the high level of similarity between the *mstA* and *mstC* transcripts (84% identity), probes corresponding to the sequences immediately downstream of the stop codons were employed to unequivocally distinguish them (see Materials and Methods for details). As can be seen in Figure 6A, irrespective of the carbon source present the *mstA* transcript is not apparent during the early phases of growth but progressively accumulates - to a greater or lesser extent - as a culture ages. By comparison, and with the exception of growth in the presence of glucose, *mstC* mRNA is transiently present during early cultivation (4–8 h) when the carbon source remains available at over 70% its original concentration. The level of this transcript subsequently declines. Some expression of *mstC* is also noted at very late culture times when the carbon source is exhausted.

The expression profile observed for the *mstC* transcript - present during the early phases of growth on glycerol and ethanol but absent upon growth on glucose - coincides with the previously reported behaviour of the glucose-repressible high-affinity glucose uptake activity in glycerol-germinating *A. nidulans* conidia [15]. *mstC* expression was therefore examined during the process of germination of two cultures of wild type conidia, one growing on glucose (a repressing carbon source) and the other growing on lactose (a derepressing carbon source) (Figure 6B). Dormant conidia (0 h) can be seen to contain *mstC* mRNA that accumulates during the first 4 h of culture in the strongly derepressing growth condition (lactose). By contrast, this basal level is rapidly depleted within 1 h when conidia are inoculated into glucose-containing medium (*i.e.* during the activation phase of conidial germination) [40], supporting the contention that the product of the *mstC* gene is the high-affinity glucose transporter detected in glycerol-germinating conidia.

### *mstC* is subject to CreA-mediated carbon catabolite repression (CCR)

Glucose repression of the high-affinity glucose uptake system has been shown to be mediated by the carbon catabolite repressor CreA [15]. *mstC* expression was thus compared between the wild type strain and two carbon catabolite derepressing mutants (*creA^d^30* and *creA^d^1*) growing in the presence of either glucose (a repressing carbon source) or galactose (a monosaccharidic derepressing carbon source). Whilst *mstC* transcription occurs in all three strains on galactose medium, this is repressed in the wild type in the presence of abundant glucose (Figure 6C). However, in the two *creA* mutants the *mstC* transcript is present on glucose. Indeed, its abundance appears to be notably enhanced, achieving levels well in excess of those seen on galactose. The *mstC* gene is thus subject to CreA-mediated repression, a characteristic also observed for the high-affinity glucose uptake system in germinating conidia.

## Discussion

In the current study two closely related genes, *mstA* and *mstC*, have been found that encode sugar permeases involved in mediating glucose transport in *A. nidulans*. Of the two, *mstC* has been shown to encode the high-affinity glucose uptake system found in wild type conidia and noted previously to be associated with the *sorA* locus [9], [15]. Indeed, compelling evidence for allelism of *mstC* and *sorA* is afforded by various data: *i)* the coincidence of their physical and genetic locations; *ii)* the occurrence of mutations in *mstC* in independent *sorA* mutant strains; *iii)* the manifestation of the *sorA* mutant phenotype upon deletion of the *mstC* gene; and *iv)* the ability of the *mstC* gene to rescue the *sorA* mutant phenotype and restore wild type sensitivity to L-sorbose.

The *A. nidulans sorA* locus was originally defined from classical genetic analysis of spontaneous mutations conferring resistance to the inhibitory effects of the paramorphogen L-sorbose on colony growth [9], [41]. The mechanism of this resistance was attributed to defects in L-sorbose transport since uptake of this sugar was noted to be dramatically reduced in *sorA* mutants whilst the utilization of a wide range of other sugars remained unaffected; the uptake of D-glucose was seen to be only slightly reduced [9]. Similar observations were also made in *N. crassa* mutants [42] (and refs therein). Further insight into the relationship between the *sorA* locus and sugar uptake has since been provided by a more recent analysis of glucose uptake kinetics [15]: of the two glucose uptake systems detected in germinating wild type *A. nidulans* conidia (*i.e.* of high and low affinities) only the high-affinity component is perturbed in a *sorA* mutant; in addition, carbon catabolite derepressed (*creA^d^*) mutant strains were found not only to constitutively express the high-affinity system but also to manifest hypersensitivity to L-sorbose. These findings are consistent with cellular entry of L-sorbose via the high-affinity glucose transporter and are in accord with previous studies in *N. crassa* reporting L-sorbose uptake to be mediated by a high-affinity glucose transporter expressed under derepressing culture conditions [43], [44]. That the adverse morphological effect of L-sorbose on colony growth of both *A. nidulans* and *N. crassa* can be reversed by glucose [9], [43] also supports this contention. Hence the function encoded by *sorA* (*mstC*) would be that of a high-affinity D-glucose transporter that also has a low affinity for L-sorbose.

Parallels observed between gene expression profiles (this work) and data from glucose uptake experiments [15] are also consistent with *sorA*/*mstC* encoding the high-affinity glucose transport system detected in germinating *A. nidulans* conidia. Both *mstC* expression and high-affinity glucose uptake occur in the presence of de-repressing carbon sources, and both are subject to glucose repression mediated by CreA. With regard to the latter it is noteworthy that CreA dysfunction results in *mstC* gene expression in the presence of glucose at a level substantially greater than that under derepressing conditions (Figure 6C). This ‘superinduction’ of *mstC* may be indicative of an inducing effect of glucose on its expression, a phenomenon already observed for genes in certain other metabolic pathways in CCR mutants [20].

*In silico* analysis (not shown) revealed the presence of three consensus CreA binding sites [45] within 100 bp upstream of the start codon and spanning a region of just 36 bp in length. However, their proximities and relative orientations (all are in the same orientation) do not correspond to the dispositions found for functional pairs of binding sites such as those regulating the genes *prnB*, *alcA* and *alcR* [45], [46], [47]. Thus, CreA repression of *mstC* may instead be effected by individual binding sites as was found for the *A. nidulans xlnA* gene [48]. The *mstA* gene, which is not subject to regulation by CreA, nevertheless has three CreA consensus binding sites present at positions −33, −302 and −316. Thus, *in silico* inspection alone of the sequences upstream of *mstC* and *mstA* is not sufficient to enable conclusions to be drawn about the regulation of these genes by CreA.

Wild type *A. nidulans* conidia germinating in the presence of glycerol exhibit high-affinity glucose uptake kinetics whilst in *sorA3* mutant strains this system is substituted by one of intermediate affinity (K_m_∼400 µM). This observation led to the suggestion that the *sorA3* mutation may cause a reduction in the glucose affinity of the high-affinity transporter [15]. In the current study however, deletion of the *mstC* gene has been shown to result in identical manifestation of both known aspects of the *sorA3* mutant phenotype, namely resistance to the toxicities of L-sorbose and 2-DOG, and substitution of high-affinity glucose uptake by a system of intermediate affinity. In the *sorA3* mutant the *mstC* gene has suffered a T to A transversion resulting in a Trp412Arg replacement in the tenth putative transmembranal domain of MstC (Figure 1). This Trp residue is described to play a role in determining substrate specificity in yeast transporters and transport capacity in the human glucose transporter GLUT1 [49]. Another *sorA* mutant, *sorA2* [9], has been found to have suffered a frameshift mutation leading to a major perturbation in MstC primary structure, yet its phenotype is the same as that observed for the *sorA3* point mutant and the *mstC* deletion mutant. These data are consistent with complete loss of the encoded uptake function in the two non-deleted *sorA* mutants rather than partial function or modification of function.

Both the primary structures and transport activities of *A. nidulans* MstA and MstC have been found to be very similar; both are strongly inhibited by CCCP and hence fall into the category of secondary active transporters, utilising membrane potential as an energy source. However, the expression profiles of the genes that encode them are quite different. The *mstC* transcript is present in dormant conidia and, with the exception of growth on glucose, its expression occurs mainly during the early phases of liquid culture. By contrast the *mstA* gene is only expressed late in mycelial culture when the carbon source is becoming exhausted. MstC and MstA may thus have derived from a common origin to play distinct physiological roles, MstC providing a high-affinity glucose uptake function during germination and the very earliest phases of mycelial growth, whereas MstA seems to provide a glucose scavenging function as a culture tends toward carbon source starvation. Regarding the latter, the analysis of substrate ranges reveals that whilst the principal substrate of both transporters is glucose, the sugars mannose, xylose and galactose could be minor substrates. In addition, it appears that the alternative substrates compete glucose uptake by MstA to a slightly greater extent than the glucose uptake effected by MstC, suggesting that MstA may act as a ‘scavenger’ of carbon sources.

Heterologous expression in yeast of four putative *A. nidulans* sugar transporter genes (*hxtB*, *hxtC*, *hxtD* and *hxtE*) under the control of the *S. cerevisiae HXT7* promoter and terminator sequences has very recently been reported [50]. Two of these, *hxtB* and *hxtD* correspond to the genes *mstC* and *mstA*, respectively, studied in the current manuscript. With the exception of *hxtD*, the other three genes were found to complement a yeast strain deleted for hexose transporters, restoring growth not only on glucose but also on fructose, mannose and galactose. Substrate competition experiments on the yeast transformants confirmed that the latter could be minor substrates, and our analysis (for *mstC*) conducted with germinating conidia is also in accord. Homologous expression of MstA (HxtD) was successful and has enabled us to gain some insight into its properties. The observation of differences in substrate specificity profiles between the heterologously expressed transporters is also reflected in our comparison between MstA and MstC. Both studies have generated *A. nidulans* deletion mutants in the transporter genes investigated and, although approached in different ways, both coincide that locus AN6669 (*hxtB*/*mstC*) encodes a high-affinity glucose transporter that confers susceptibility to toxic analogues of D-glucose. Direct measurement of sugar affinities made in the case of the heterologously expressed *A. niger* orthologue MstA [51] also yielded a Km value for glucose very similar to that found for MstC and in addition showed that certain other sugars could be minor substrates. Interestingly, the sugar transporter gene deletions carried out to date in *A. nidulans* (this work) [16], [50] and *A. niger* [51] failed to yield clear morphological phenotypes thus indicating functional redundancy and hence the existence of a number of transporters capable of transporting glucose.

The analysis reported in this study along with that of dos Reis *et al* [50] and the work on the low-affinity transporter encoded by *mstE* [6], bring to five the number of *A. nidulans* glucose uptake systems for which functional characterisation has been achieved. A phylogenetic analysis of Eurotial homologues corresponding to these transporters (as well as the hypothetical transporter HxtA) is presented in Figure S1. Whilst the MstE, MstA/MstC and HxtE groups are represented in many species, only a limited number of species appear to have MstD (GenBank AM168452) - HxtC in [50] - or HxtA group members. Conversely, two sequenced Aspergillus species (*A. glaucus* and *Eurotium rubrum*) lack members in all groups except MstE. Given the evidence that MstA/C and HxtE are high-affinity glucose transporters [50] (and the current work) this suggests that other high-affinity glucose transporter genes have yet to be identified. In this regard the question remains as to the identity of the gene that encodes the system of intermediate affinity observed in *sorA* mutants [15]. Both the expression patterns and glucose affinities of MstA and MstE [16] exclude these as possible candidates for substituting MstC. Indeed, a *sorA3 ΔmstE* double mutant germinating on glucose shows *sorA3* single mutant uptake kinetics [16], and the same is true for a *ΔmstA ΔmstC* double mutant germinating on glycerol (this work). Although the genetic basis of this transporter is unknown, its expression may be negatively correlated with the activity of the high-affinity system and only operative in its absence. Further studies are required to identify other glucose transporters, specifically that responsible for intermediate-affinity glucose uptake.

## Supporting Information

Figure S1 **Unrooted phylogenetic tree of sequenced Eurotial genome orthologues of the six sugar transporters studied to date (encoded by** ***A. nidulans*** **loci: AN1797, AN5860, AN6669, AN6923, AN8737, AN10891).** Evolutionary history was inferred using the Maximum Likelihood method, and the numbers of replicate trees in which the associated sequences clustered together in the bootstrap test (50 replicates) are shown next to the branches. Branch lengths correspond to the mean number of substitutions per site. The proteins used to define each group are highlighted in yellow. Where known, genome locus identities are given; ‘Corr’ indicates that the gene model was corrected; the correct gene models for MstD (AN10891) and HxtA (AN6923) were used and are found in their GenBank accession data, numbers AM168452 and AJ535663, respectively.
(TIF)
