# Supplementary figures and images for: High-Affinity Glucose Transport in Aspergillus nidulans Is Mediated by the Products of Two Related but Differentially Expressed Genes
Source: PLoS One. 2014 Apr 21;9(4):e94662. doi: 10.1371/journal.pone.0094662 (PMC3994029; doi:10.1371/journal.pone.0094662)

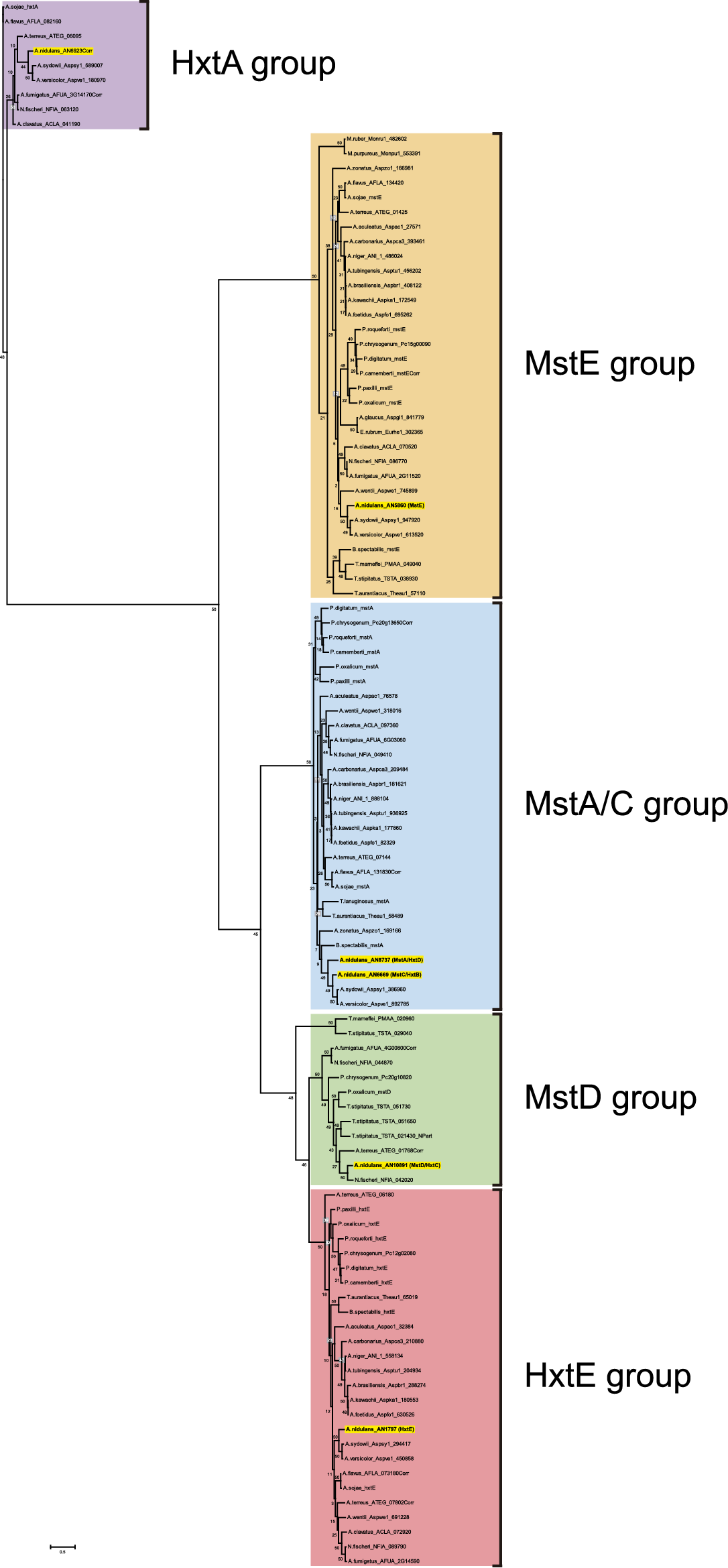

Supplement: Figure S1 — Unrooted phylogenetic tree of sequenced Eurotial genome orthologues of the six sugar transporters studied to date (encoded by A. nidulans loci: AN1797, AN5860, AN6669, AN6923, AN8737, AN10891). Evolutionary history was inferred using the Maximum Likelihood method, and the numbers of replicate trees in which the associated sequences clustered together in the bootstrap test (50 replicates) are shown next to the branches. Branch lengths correspond to the mean number of substitutions per site. The proteins used to define each group are highlighted in yellow. Where known, genome locus identities are given; ‘Corr’ indicates that the gene model was corrected; the correct gene models for MstD (AN10891) and HxtA (AN6923) were used and are found in their GenBank accession data, numbers AM168452 and AJ535663, respectively. (TIF) [file pone.0094662.s001.tif]
